# Barriers, facilitators, and opportunities for hospital antimicrobial stewardship in low and lower middle - income countries in the Eastern Mediterranean region: results from a mixed methods study

**DOI:** 10.1186/s13756-025-01625-3

**Published:** 2025-10-14

**Authors:** Nour Shamas, Elizabeth Tayler, Miriam Holm, Hala Amer, Shaffi Fazaludeen Koya

**Affiliations:** 1 World Health Organization Regional Office for the Eastern Mediterranean, Cairo, Egypt; 2 Antibiotic Policy Unit City, St George’s University, London, UK; 3 Independent Consultant, Beirut, Lebanon

**Keywords:** Antimicrobial resistance, Antimicrobial stewardship, Implementation, Facilitators, Barriers, Eastern Mediterranean region

## Abstract

**Background:**

Antimicrobial stewardship programmes (ASP) are crucial for mitigating antimicrobial resistance (AMR), a growing threat in the Eastern Mediterranean region (EMR) where conflict, instability, and economic challenges hinder health systems. EMR specific barriers of antimicrobial stewardship (AMS) remain under documented.

**Methods:**

A mixed methods study was conducted to explore barriers and opportunities for ASP implementation in EMR focusing on low (LIC) and lower middle-income countries (LMIC) with relatively stable policy environments and demonstrated interest, capacity, and infrastructure for stewardship. We used literature review, semi-structured interviews of experts, and a stakeholder consultation.

**Results:**

Seven key themes emerged: One, AMS implementation capacity varies across the region, necessitating tailored approaches. Two, the limited availability of expertise requires urgent upscaling of knowledge and capacity. Three, mentorship and the development of centres of excellence is needed. Four, existing AMS tools should be enhanced, disseminated, and occasionally, adjusted to local needs. Five, the lack of a sustainable platform for networking impedes collaboration. Six, accreditation and national level mandates for hospital AMS can support scale-up. Seven, expanding research is critical to inform local evidence-based action.

**Conclusions:**

Several components of ASPs are achievable in the EMR using existing resources with targeted support from local and identified regional and global partners.

**Supplementary Information:**

The online version contains supplementary material available at 10.1186/s13756-025-01625-3.

## Background

Antimicrobial resistance (AMR) causes substantial harm at various socioeconomic levels in the society [1] with an estimated associated 4.71 million deaths in 2021 [2]. Although the causes of AMR are multifactorial and include systemic structural barriers such as lack of access to clean water and sanitation and variable infection prevention and control practices [1]the most significant driver of AMR is inappropriate antimicrobial use. Global efforts to promote the responsible use of antimicrobials are progressing, however there are several barriers to the success of such programmes [3–5].

The World Health Organization (WHO) defines antimicrobial stewardship (AMS) as organizational or system-wide health-care strategies to promote the appropriate use of antimicrobials through the implementation of evidence-based interventions [6]. Although most antimicrobials are used in primary care [7], AMR transmission is expected to result from extensive antimicrobial use in hospital settings [8], As relatively more resources are available for inpatient AMS [9] if available, AMS tends to be more commonly implemented in hospital settings.

The Eastern Mediterranean Region (EMR) of the WHO includes 22 countries in Asia, the Middle East, and North Africa. Global Antimicrobial Resistance and Use Surveillance System (GLASS) data and studies suggest high prevalence of drug resistance and inappropriate use in the EMR [10, 11]. Available data also show higher per capita antibiotic consumption rates in the region compared to the global average in 2018. Although AMR national action plans have been developed in most countries, stewardship activities are suboptimal [12]. The WHO Regional Office for the EMR (WHO-EMRO) developed a stewardship framework in 2024 [13] to guide AMS in hospital and primary care settings, and member states have recently endorsed the decision to collaborate and accelerate the response to AMR [14], however implementation faces several challenges.

In terms of AMS capacity, EMR countries fall into three groups. The six high-income Gulf Cooperation council (GCC) countries where progress is tangible and have capacities to scale up hospital AMS nationally [15, 16]. Some are fragile, conflict-affected, and vulnerable (FCV) countries where AMR is a major concern [17, 18] but where hospital level interventions are harder to implement and efforts are limited to specific settings [17, 19] as other urgent crises tend to take precedence. The third group includes Low income (LIC) and Lower middle -Income countries (LMICs) which report the highest per capita antibiotic consumption compared to GCC countries and FCVs.

Evidence or documentation of implementation experiences and barriers in the region is limited [15]. Therefore, we decided to conduct a study to summarise the existing literature from the region and identify main barriers, facilitators, and opportunities in implementing AMS in hospitals in low- and middle-income countries in EMR. The study focused on LICs and LMICs where the risks of inaction are high, and where there is a relatively stable policy environment as well as interest, capacity, and infrastructure for AMS.

## Methods

We used a mixed methods approach to conduct the study, which included a rapid literature review, in-depth interviews with experts, and a stakeholder consultation.

First, we conducted a focused rapid review following the WHO practical guide for rapid reviews to strengthen health policy and systems [20], with the following question: *What is the current literature on barriers and facilitators in implementing AMS in hospitals in low and lower-middle income countries in EMR.* We searched PubMed on 15 February 2024 using a structured search strategy (Supplement S1) and reviewed websites of WHO and other relevant agencies working in AMR and AMS in addition to snowballing of references to identify pertinent literature in English for the period 2017—2023. We used The Preferred Reporting Items for Systematic reviews and Meta-Analyses (PRISMA) checklist as relevant to rapid reviews to guide reporting (Supplement S2). We used a “descriptive-analytical” method to extract information [21] applying a common framework to all the papers included to collect standard information on key issues and themes using pre-defined table (author, year, country, methods used, key facilitators and barriers). One author (NS) extracted the information, and after a joint review by NS and SFK, a narrative synthesis was written.

Second, we conducted semi-structured in-depth interviews with experts in the field of AMS and AMR, including those from regional organizations that could support capacity building in these fields. The experts were selected through a review of available literature and websites related to AMS and AMR, snowballing technique, and through prior knowledge and collaboration of the authors with regional experts and organisations. We sent them an email, and upon receiving consent, we shared the interview questions by email (Supplement S3) ahead of the online recorded interview. In general, the questions were on facilitators and barriers to successful scale up of hospital AMS, but also probed for opportunities and tools that can help fill the gaps. Thematic saturation was reached using a mixed deductive–inductive approach. We began with a deductive framework based on literature and study objectives, then incorporated inductive analysis to capture emerging themes. Saturation was considered achieved when no new themes or subthemes emerged and existing categories were sufficiently detailed. The focus of the interviews with experts from organizations was to identify their current efforts and available resources related to hospital AMS scale up that can be considered for EMR, and to identify collaboration potential. All interviews were recorded, transcribed, and key themes were identified and tabulated with narratives by one author (NS) [22].

Third, we shared the key findings from the rapid review and expert interviews with experts from WHO, ministries of health, WHO collaborating centres, and other relevant agencies during a stakeholder consultation meeting. The discussions were captured by rapporteurs and were analysed by thematic subgroups by one author (NS). All findings from the review, interviews, and consultation were reviewed by all the authors.

This study did not require formal ethics approval as it involved interviews with key experts who participated in their professional capacity and did not collect any individual data. All participants were informed about the purpose of the study and provided consent over email to participate and to share their insights in the report.

## Results

### Rapid review

Our literature search identified 14 studies in PubMed [16, 23–35], 11 of which discussed facilitators and barriers to implementing AMS in the EMR, and 3 of which were excluded as they were out of scope [26, 34, 35]. An additional study was identified from the references [36]. These include one systematic review, one literature review, three cross sectional surveys, and seven qualitative studies. In addition, WHO has published several resources relevant to AMS which are included in the AMR resource pack (Supplement S4) [37]. 

### Interviews

Of 42 potential experts contacted and invited for interviews, 37 responded and 36 agreed to the interview. One expert who declined the interview suggested a replacement and an additional expert was suggested by one of the interviewees, both of whom agreed to be interviewed. As several organizations nominated multiple experts for the interviews, we finally conducted 38 interviews (64.3% of women) with 56 individuals including 12 independent experts, 18 professional organizations, staff from Southeast Asia Regional WHO office (SEARO) and 5 WHO Country Office staff (Table 1). After all the interviews were completed, the findings were summarised and shared with the interviewees for review and confirmation.

**Table 1:** Summary of geographical representation of interviewees

Regions	Number of interviewed experts (% women)	Number of organizations	Number of interviewed experts representing organizations** (% women)	Total number of interviewed experts (% women)
EMR representatives: - Experts - WCO^1^	8 (87·5%) NA*	4 5	12 (58.3%) 11 (72·7%)	31 (70·9%)
Outside EMR International representatives				
- *Experts* - *SEARO*^*2*^	4 (50%) NA	14 1	19 (63·2%) 2 (50%)	**25 (56%)**
*Total*	**12 (75%)**	**24**	**44 (61**·**4%)**	**56 (64**·**3%)**
*Regions*	Number (% women)		Total number of interviewed experts (% women)	
*EMR representatives*: - *Experts* - *WCO*^*1*^ - *Experts representing organisations*	- 8 (87·5%) - 11 (72.7%) representing 5 WCO - 12 (58.3%) representing 4 organisations		**31 (70**·**9%)**	
*Outside EMR* *International representatives* - *Experts* - *SEARO*^*2*^ - *Experts representing organisations*	- 4 (50%) - 2 (50%) - 19 (63·2%) representing 14 organisations		**25 (56%)**	
*Total*			**56 (64**·**3%)**	

^1^WCO: WHO country offices, SEARO: WHO Regional Office for South-East Asia

*Not applicable **Some organisations had several experts available at the interview

Of the 18 interviewed organisations, 16 have had experience providing online education, 13 have provided in-person or hybrid format education, eight have supported networking, eight have an aspect of advocacy, six have worked on policy, six have provided mentorship opportunities, and six have had experience with AMS implementation support. Only four organisations have provided technical support, and four provide AMS tools. Lastly, only one organization is supporting accreditation explicitly. We have summarized the interviewed organizations according to their key areas of expertise and engagement in Supplements S5 and S6. Supplement S7 presents illustrative quotations organized by thematic categories.

### Stakeholder consultation meeting

The consultation meeting was conducted in hybrid mode on the 5th and 6th of March 2024 with 37 attendees. The participants included a selection of interviewed experts from international and regional agencies, experts from the ministries of health, and several units from within the WHO EMRO including AMR, medicines, health systems, child and adolescent health, partnerships, health emergencies, science information and dissemination, patient safety and quality, and communicable diseases. Meeting findings and recommendations were then added to the overall interview and literature search findings and shared with all participants through email for final feedback.

### Key findings

#### Rapid review

The key factors facilitating hospital AMS included the availability of trained healthcare workers, effective facility leadership support, and the availability of regulation (in different forms). The common identified barriers include the lack of leadership or stakeholder support, lack of training resources, lack of regulatory enforcement, and limitations in human resources (infectious disease or AMS trained physicians or pharmacists, microbiologists), financial resources, and tools (antimicrobial susceptibility testing (AST), AMS intervention tracking software, and information technology). The systematic review conducted by Hashad et al. evaluated 17 ASP implementation studies from high income Gulf cooperation council countries. This review highlighted that even in high income settings, lack of funding and dedicated staff are barriers to the success of AMS [16]. Table 2 summarises the key findings from 12 studies included in our review.

**Table 2:** Barriers and facilitators to AMS implementation in EMR based on the rapid literature review

Articles	Study methods	Country or region	Barriers	Facilitators
Alghamdi et al., 2019 [26]	Semi-structured interviews with healthcare professionals	Saudi Arabia	• Lack of enforcement of policies and guidelines from the Ministry of Health and hospital administration • Disintegration of teams • Poor communication • Lack of recruitment/shortage of ASP team members • Lack of education and training • Lack of health information technology (IT) capacity • Physicians’ fears and concerns in relation to liability of adoption of ASPs.	• Not mentioned
Baraka et al., 2019 [28]	Survey about clinicians’ (physicians, pharmacists and nurses) previous experience with AMS	Eastern province of Saudi Arabia	• Lack of internal policy/guidelines and specialized AMS information resources. • Lack of administrative awareness about AMS programs • Lack of personnel • Time limitation • Limited training opportunities • Lack of confidence • Financial issue or limited funding • Lack of specialized AMS information resources	• Good formulary management • Provision of real-time feedback • Provision of didactic education (lectures from infectious disease specialists and pharmacists) • Availability of online AMS resources and clinical guidelines should be accessible • Availability of AMS team • Leadership support • IT department support • Time and incentives/funding • Availability of pathogens and antimicrobial susceptibility test (AST) results
Nasr et al., 2019 [32]	Interviews with pharmacist	Qatar	• Electronic system errors	• Collaboration and communication among teams • Compliance with guidelines • Documentation of interventions by clinical pharmacists in medical charts
Hashad et al., 2020 [16]	Systematic review based on selection of studies	Gulf Cooperation Council states	• Lack of dedicated staff • Heavy workload • Lack of Funding	• Physician support • Availability of information systems • Education
Mehtarpour et al., 2020 [27]	Semi-structured interviews with managers from the Ministry of health, Iran veterinary organization, the national professional associations and researchers	Iran	• The migration of infectious patients • Trafficking of medicine and livestock from neighbouring countries • The imposed sanctions	• Knowledge transfer • Facilitation in policy agenda setting • Formulation and implementation process • AMR monitoring
Atif et al., 2021 [22]	In-depth interviews with medical doctors.	Pakistan	• Poor knowledge of doctors regarding ASP • Non-existence of hospital antibiogram • Lack of rules for the safe use of antibiotics	• Development of guidelines for the use of antibiotics • Strict legislation regarding use of antibiotics • Active participation of healthcare professionals • Awareness program among general public about the use of antibiotics
Orubu et al., 2021 [24]	Survey by prescribers/physicians and pharmacists	Yemen	• Routine use of AST to guide the choice of antimicrobial agent was limited • Lacked prescribing guides	• The deployment of reliable, affordable, quality rapid diagnostics, and AST kits • Compulsory continuing education emphasizing the use of AST to guide prescribing • Patients’ awareness programs • Capacity increase and training for laboratory personnel • Logistics support
Sayegh et al., 2021 [31]	Descriptive cross-sectional survey by physicians	Lebanon	• Physicians lack of compliance with hospital guidelines and antibiotic prescribing policies • Minimal support of the MOPH and absence of regulation • Absence of national approved guidelines • Lack of training and education on antimicrobial use • Lack of support from the medical staff • Lack of leadership to promote antimicrobial stewardship • Lack of financial incentives to the ID physician/ pharmacist to initiate the program • Lack of support from administration or department heads • Infectious disease physician shortage • Perception that there is insufficient evidence that the hospital would benefit from AMS	• Not mentioned
Rizk et al., 2021 [35]	Review article focused on the impact of COVID-19 on AMS and AMR	Countries of the Arab League	• Poor quality of generic medicines • Sub-optimal laboratory facilities • Antimicrobial shortages • Lack of education	• Not mentioned
Hashad et al., 2023 [23]	Semi-structured interviews conducted with ASP stakeholders	UAE	• Blame culture • Complexity of ASP implementation • Shortage of expert personnel	• External policy requirements (both national and international) • Leadership support • Stakeholders’ engagement • Collaborative culture • Effective communication • Forward planning
Salem et al., 2023 [30]	In-depth interviews with physicians, paediatricians, and clinical pharmacists	Egypt	• Time Constraints • Lack of Awareness of AMS • Difficulty in monitoring of ASP • Lack of available AMS guidelines.	• Having adequate human and non-human resources • Having clearly defined goals and objectives and precise definitions of the role of key stakeholders • Education and training
Shallal et al., 2023 [29]	ASP focused Workshops attended by hospital directors, ASP team members, and IPC officers	Amman, Jordan	• Lack of human resources • Lack of financial resources • Inadequate regulation for prescription antibiotic sales • Insufficient stewardship or infection control training • Limited laboratory capacity • Lack of technical expertise • Inadequate internal communication between administration and staff • IT support insufficient and manual work	• Not mentioned

## Interviews and consultation

We identified seven key themes through the interviews and stakeholder consultation meeting. These are: AMS programme (ASP) systems and structure, training and education, mentorship and centres of excellence, tools, networking, accreditation, and research.

### ASP systems and structure

There are substantial variations in AMS resources, capacity, and structure between and within countries. Some countries do not have enough trained core AMS members such as infectious disease physicians, pharmacists and microbiologists. In other settings, clinical pharmacists and pharmacists are leading ASP implementation and training. Even when expertise is available, poor communication, strong hierarchies, and lack of leadership support may impede implementation. Since AMS is multidisciplinary, members of the healthcare team (physicians, pharmacists, microbiologists, and nurses) need to be empowered. Although ASPs in some countries are led by Infectious Disease (ID) physicians, this is not always feasible.

With existing hierarchies in healthcare, a lack of clear roles and rules of engagement with prescribers can be discouraging for pharmacists and lead to a diminished impact in hospital ASPs as pointed out by our interviewees. Experts also pointed out the need for clearly defined terms of reference (ToR) for the AMS team and its members thus creating a sense of structure and system for the ASP. The availability of a national AMS committee was cited as a potential facilitator when present, although not a prerequisite to starting and scaling up AMS within any facility. Experts identified medication shortages and lack of integration of AMS into infection control programs or medication safety programmes as barriers to AMS implementation. Experts identified national and hospital medication formularies, antimicrobial use or infectious disease treatment guidelines, and AMR surveillance as facilitators to scaling up AMS.

### Training and education

A key factor that came up in the interviews was the disparities in the level of knowledge and understanding of the essential concepts of AMR, microbiology, pharmacology, and appropriate prescribing and use across HCWs. Additionally, the availability of specially trained workforce including ID physicians and clinical pharmacists, among others, with training and expertise in AMS and with expertise in leading AMS programmes is limited in most settings.

### Undergraduate or pre-service education

Explicit interest emerged in the need to optimise undergraduate medical education for all cadres to increase the awareness and sense of accountability of future health workforce in AMS governance, interventions, and their roles. WHO’s curricula guide and assessment tools [38] are useful tools and although some universities are interested in implementing the full exercise, there was an interest in a more direct one-size-fits-all starter kit for implementing curriculum adjustments (such as a slide set of major concepts: essential concepts of AMR, microbiology, pharmacology, and appropriate prescribing and use, and the need for multidisciplinary approach in all these efforts).

### In-service education and continuous professional development (CPD)

Interviewees expressed that HCWs of all different specialities need sensitization and training on appropriate antimicrobial prescribing and use, particularly for prescribers and pharmacists who have just qualified. This can be in-service training/ seminars/ workshops for public sector providers or CPD in general. The WHO AWaRe antibiotic book and the mobile application [39] are identified as effective tools for this training.

### Specialised training on AMS program components, implementation, and leadership

HCWs who may lead hospital AMS efforts such as physicians, pharmacists, microbiologists, and possibly nurses need specialised AMS training to structure and implement the program. It was recommended that these programmes should cover the basics of leadership and programme management, including intervention prioritisation, patient safety and quality improvement concepts in addition to ensuring advanced concepts in AMR, microbiology, pharmacology, and appropriate prescribing and use. The curriculum should also include content to improve effective collection, analysis, presentation, feedback, and use of data, and should aim for “team coaching”.

### Mentorship and centres of excellence

A significant barrier to scaling up AMS is the lack of mentorship and longitudinal support for AMS champions. There are few centres with experience in running ASP for substantial period, and therefore there are very few training centres. Even when AMS training was available, it was short-lived whereas practical AMS problem solving requires significant peer-to-peer learning and mentorship.

### AMS tools

AMS tools, including digital tools, can improve efficiency, scale-up ASPs, and reduce duplication of work. These tools may help with organizing data, reviewing prescription, providing feedback, and assessing progress. Digital tools may include clinical decision support systems and mobile applications and chatbots for guiding prescriptions and dashboards for monitoring ASPs. Although several tools exist, absence of a centralized repository make it difficult for new users to make the best use of these resources.

In addition to mapping available tools, there is a need to develop and customize these tools to local requirements.

### Networking

Networking between institutions, practitioners, and experts is a significant facilitator to supporting the scale up of AMS. Professional society involvement was highlighted as a driver for increased awareness leading to grassroot initiatives. This is particularly important when national governance is not yet actively engaged in mandating or supporting AMS. There are possibilities for these bottom-up AMS initiatives in the form of centres of excellence or regular meetings of local experts to lead to national engagement in pushing the AMS agenda.

### Accreditation and regulation

Many interviewees noted the importance of having local or international hospital accreditation standards to support AMS implementation. Although insufficient by itself and may be difficult to enforce in many situations, this can be a powerful driver for leadership support and resource allocation especially when they are part of a broader quality improvement. The subject of regulation of antimicrobial use through mandating hospital AMS at a national level was also considered a facilitator.

### Research

The literature review and expert interviews showed the lack of local and regional research focused on AMS and AMR. Research on antimicrobial use and resistance, prescription practices and behaviours, use of diagnostic tools, as well as evaluation of interventions through implementation or operational research will help improve and tailor regional AMS efforts.

## Discussion

This study identified key challenges and opportunities to scale up AMS in hospitals in LICs and LMICs in EMR. Although some AMS efforts are underway in some settings, scale up in EMR requires a robust plan for providing technical support to countries. There are seven important messages that emerge from this study which are discussed below and Table 3 summarizes the key recommendations to scale up hospital AMS in EMR.

First, tailored approaches are required to implement sustainable AMS programs in the region with many countries experiencing shortage of HCWs resulting in lack of time and capacity to focus on optimising use of antimicrobials. Considering the time needed to develop tailored approaches we need to balance preset packaged, standardized, and reproducible interventions that are scalable with minimal tailoring to context. The minimum set of interventions in a facility should be adjusted to the resources available and the complexity of case management.

ASPs in the region should be modelled on available resources with flexibility in the scope of practice of relevant professionals. The ASPs could be led by an Infectious Disease (ID) physician, an AMS trained non-ID physician, a clinical pharmacist or trained pharmacist, a clinical microbiologist, a nurse, or a team. Clearly defined ToRs can facilitate multidisciplinary collaboration and improve accountability and governance. AMS training modules should include the importance of multidisciplinary teams, behavioural approaches, and communication. Programs can be housed in any department or as standalone but need to have a strong interface and collaboration with IPC and microbiology department. Whenever available, hospitals may consider using the expertise of behavioural scientists [40], health promotion experts, quality and patient safety experts [41], and data experts or epidemiologists in improving their ASPs. For coherent scale-up across a country, there is a need for national leadership to monitor and manage the process.

Two, an urgent upscaling of knowledge across different levels of training is needed. This requires the introduction of minimum requirements in pre-service training, besides continued in-service education and training. Additionally, there is a need for dedicated training to develop a specialist cadre to lead AMS programmes. These trainings can target ID physicians, clinical pharmacists, nurses, and public health specialists/epidemiologists and enable them to carry out relevant clinical, public health and program management and leadership functions.

All training should cover soft skills related to communication, programme management, quality improvement and leadership principles. Existing models of training by different agencies provide several options: online, face-to-face, or hybrid; training individuals versus AMS teams together; with duration ranging from a few hours to months, with or without projects and mentorship. Successful massive open online courses (MOOCs) and training available online [42–47] (see supplement S6) can be scaled-up and tailored to the local contexts. Contextualizing may include the addition of cases from the local context or specific local tools.

**Fig. 1:**
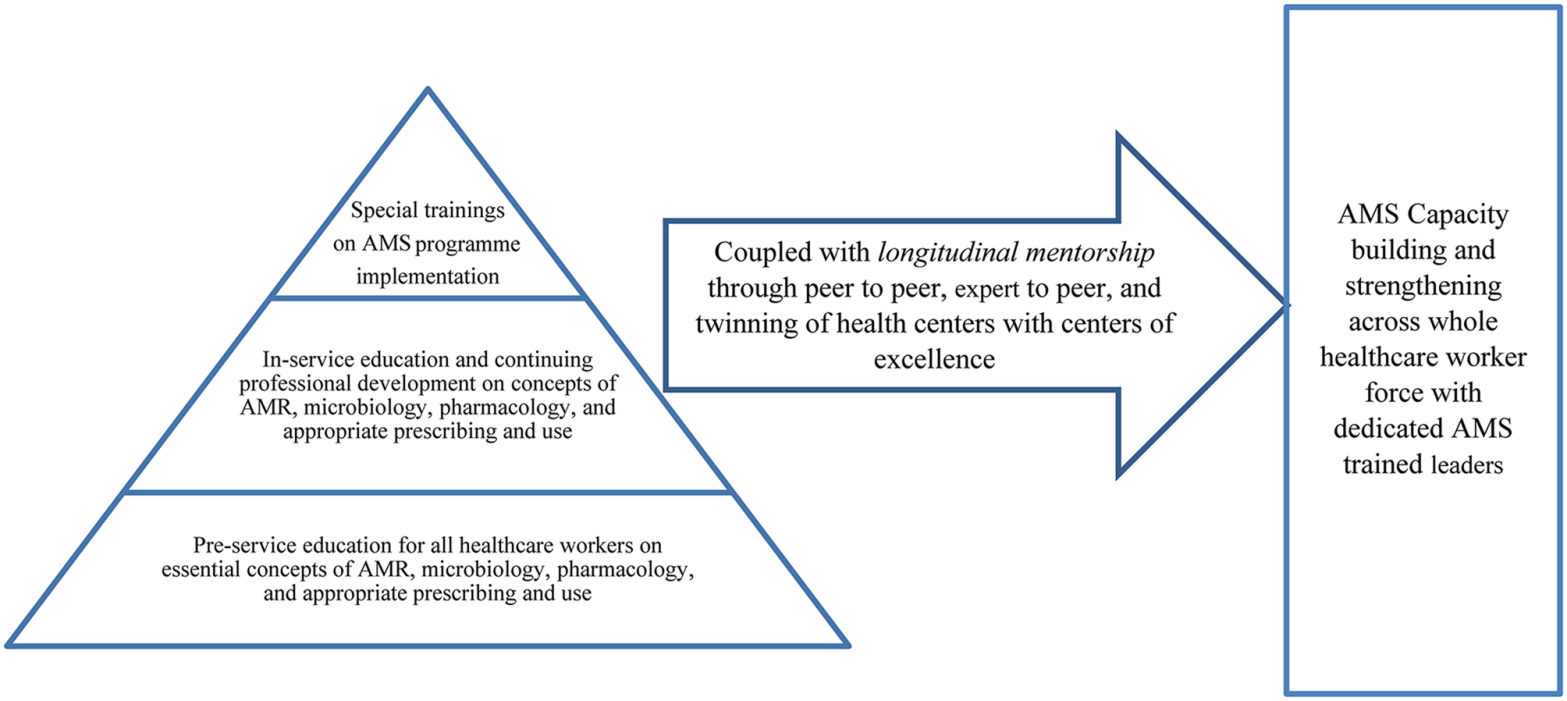
Suggested approach to scaling up AMS knowledge and capacity in EMR

Third, there is a need for a longitudinal mentorship programme to help AMS teams with real-world implementation exposure, by twining hospitals with centres of excellence (COE). Studies like the one by Moehring et al. [48] have demonstrated the merits of such collaboration on reducing antimicrobial consumption. Figure 1 summarises the suggested approach for training and mentorship in the region.

Four, provision of tools to support implementation could speed up scaling up hospital AMS [16, 25, 28, 29, 32] by mapping of tools and making them available in a public repository. In addition to WHO tools, these may include: antimicrobial guidelines developed by various societies and organizations, pre-set customizable intervention templates to build business cases and project charters with clear objectives and key performance indicators (KPIs), data collection tools [49–52], education materials and ready-to-use job aids (flip charts or posters) materials [49, 53], electronic phone or web applications to support prescription decision making [39, 50], electronic programmes which incorporate electronic health/medical records systems to help review prescriptions and suggest modifications on a nearly real time basis [51], and digital programmes that can auto-calculate antibiotic use patterns including using a defined daily dose (DDD) matrix, and generate indicators like Days of Therapy/other KPIs) [52]. Availability of these tools does not ensure implementation but facilitates it. Guidelines should be evidence based and aligned with the WHO AWaRe Antibiotic book [40] and should be accompanied by capacity building to ensure the optimal use of hospital or local surveillance data.

Five, experts and organizations cited the lack of a sustainable long-standing platform to support mentorship and development of professional relationships and collaboration. Although in recent years there has been some increase in the number of conferences and networking events in the region, very few conferences, organisations, or networks explicitly discuss AMR regionally, similar to what He et al. identified in Asia [54]. Although the impact of networking on AMS has not been widely studied, a systematic review identified the advantages of networking on the quality and safety of patient care [55]. There is a need to identify, enlist, and engage relevant AMS-related professional societies in each country.

Six, AMS accreditation was widely cited as a facilitator to ASP implementation in the EMR, although others have reported that accreditation may become a box-ticking exercise and lead to superficial compliance [56]. Some of the existing hospital accreditation standards include AMS elements [57]. Standalone ASP accreditation programmes do exist although rare [58, 59]. The accreditation requirements have given AMS teams the opportunity to garner human resource, governance support, and sometimes financial support. In some countries, such as Egypt, AMS is mandated by the government for specific hospitals. With a people centred approach [60], AMS efforts should be aligned with IPC and patient safety and quality as these are drivers of change– and similar integration into other accreditation standards may also be considered. For example, surgical prophylaxis indicators could be added into surgery and anaesthesia key performance indicators and accreditation requirements, supporting AMS efforts in a multidisciplinary approach.

Seven, regional AMR and AMS data collection and research capacity needs support. There are limited studies on AMS in the region and as interviews revealed the availability of data on antibiotic use is limited in most countries. This is similar to the findings in the systematic review by Ababneh et al. in the region which showed paucity of published data [15]. In addition to improving ASP readiness, capacity building focused on data collection and use can support the generation of local and regionally relevant evidence for action.

This study has some limitations. We did not conduct a full-scale systematic review for this study. However, for programmatic recommendations, a rapid focused review is sufficient. We conveniently selected the interviewees and organizations to reflect regional expertise in AMS and AMR from among those with the mandate and experience. Therefore, we might not have captured perceptions from other relevant stakeholders like professional associations and professional licensing bodies who may have a role in wider AMS interventions. Although only a single author conducted the data extraction and analysis, all the authors discussed the findings in a structured way to avoid the risk of subjective interpretation. Finally, the recommendations are prepared considering the regional needs. However, since the situations in low- and middle-income countries globally may not be substantially different, we believe many of these recommendations may be relevant globally.

**Table 3:** Suggested interventions to support scale up of hospital AMS in EMRO

Recommendation
1. **AMS programme structure** 1.1. Develop a set of models for AMS programme structures considering various scenarios of human resources available (availability of ID physicians, clinical pharmacists, or clinical microbiologists) and complexity of the clinical mix (secondary vs. tertiary vs. specialist hospitals)
2. **Training and education:** 2.1. Pre-service: Incorporate IPC and AMS training into healthcare worker force undergraduate education for nurses, physicians, pharmacists, midwives, and dentists, as well as general AMR education and awareness in all undergraduate training. 2.2. Pre-service and in-service: develop training for new prescribers on antimicrobial use and the roll-out of the WHO book. 2.3. In-service: ensure accessible training and education modules on AMS for physicians and pharmacists 2.3.1. In addition to typical AMS courses, the curriculum should include soft skills training, quality improvement and project management, and integrate AMS from a patient safety perspective
3. **Mentorship and centres of excellence**: Harness available mentorship opportunities for longitudinal guidance and pairing of institutions 3.1.1. Identification and highlighting available centres of excellence through a semi-formal process 3.1.2. Develop a list of regional experts interested in mentorship and train them on providing AMS mentorship 3.1.3. Twinning of interested hospitals with centres of excellence
4. **Accreditation and Regulation**: 4.1. Recommend incorporation of AMS into national accreditation standards with considerations for variability of expectations based on the setting (i.e.: could develop 3 layers of AMS accreditation with a minimum requirement) 4.2. Collaborate with national professional bodies to develop requirements for continuing education on AMS
5. **Operational tools**: 5.1. Develop templates for building a business case for AMS for high resource hospitals and low resource hospitals 5.2. Develop templates for AMS interventions 5.3. Tools for AMS program management and audit 5.4. Develop digital systems that can generate reports on antimicrobial use. 5.5. Optimize use of diagnostics to optimize antimicrobial use and reduce waste in healthcare worker time and resources
6. **Networking**: 6.1. Promote the use of the community of practice website and evaluate engagement and need for adjustment of the platform or supplementation with alternative platforms 6.2. Support the development of networking platforms though available resources and through nudging local and regional institutions
7. **Support regional research efforts to improve implementation and scale up** 7.1. Identify regional research priorities based on the WHO AMR Research priorities list 7.2. Design tools for simple studies in behavioural science in the region

## Conclusions

The study identified some important barriers in the implementation of hospital ASPs in LICs and LMICs in the EMR. Several solutions that emerged from this study may not require substantial investment. Considering that most of the recommendations closely align with two of the three WHO Regionals flagship initiatives–on access to medicines and diagnostics and skilled health workforce [61], it is important to build partnerships between organizations with resources and interest to scale-up some of the low-cost interventions. There should be operational and implementation science research to understand the implementation challenges and to identify the most impactful and cost-effective interventions.

## Supplementary Information

Below is the link to the electronic supplementary material.

Supplementary Material 1

## Data Availability

No datasets were generated or analysed during the current study.

## Abbreviations

AMR: Antimicrobial resistance
AMS: Antimicrobial stewardship
ASP: Antimicrobial stewardship programme
COE: Center of excellence
EMR: Eastern Mediterranean Region
HCWs: Healthcare workers
IPC: Infection prevention and control
LIC: Low-income country
LMIC: Lower middle-income countries
ToRs: Terms of reference
WHO: World Health Organization
