# Supplementary material for: Barriers, facilitators, and opportunities for hospital antimicrobial stewardship in low and lower middle - income countries in the Eastern Mediterranean region: results from a mixed methods study
Source: Antimicrob Resist Infect Control. 2025 Oct 14;14:119. doi: 10.1186/s13756-025-01625-3 (PMC12523090; doi:10.1186/s13756-025-01625-3)
Supplement: Supplementary file 1 — Supplementary Material 1 [file 13756_2025_1625_MOESM1_ESM.docx]

**Supplement S1: Search string used in PubMed on 15 February 2024**

((facilitators[Title/Abstract]) OR (Barriers[Title/Abstract]) AND ("Antimicrobial Stewardship"[Majr] OR "Antibiotic Stewardship" [Text Word])) AND ("Afghanistan"[Text Word] OR "Bahrain"[Text Word] OR "Djibouti"[Text Word] OR "Egypt"[Text Word] OR "Iran"[Text Word] OR "Iraq"[Text Word] OR "Jordan"[Text Word] OR "Kuwait"[Text Word] OR "Lebanon"[Text Word] OR "Libya"[Text Word] OR "Morocco"[Text Word] OR "Oman"[Text Word] OR "Pakistan"[Text Word] OR "Qatar"[Text Word] OR "Saudi Arabia"[Text Word] OR "Somalia"[Text Word] OR "Sudan"[Text Word] OR "Syria"[Text Word] OR "Tunisia"[Text Word] OR "UAE"[Text Word] OR "Palestine"[Text Word] OR "Yemen"[Text Word]) AND (2017:2023[pdat])

**Supplement S2: PRISMA 2020 checklist: Barriers and opportunities for hospital antimicrobial stewardship in low and lower middle - income countries in the Eastern Mediterranean region: results from a mixed methods study**

| **Section and Topic** | **Item #** | **Checklist item** | **Location where item is reported** |
| --- | --- | --- | --- |
| **TITLE** | | |  |
| Title | 1 | Identify the report as a systematic review. | 5 (as rapid review) |
| **ABSTRACT** | | |  |
| Abstract | 2 | See the PRISMA 2020 for Abstracts checklist. | N/A |
| **INTRODUCTION** | | |  |
| Rationale | 3 | Describe the rationale for the review in the context of existing knowledge. | 5–6 |
| Objectives | 4 | Provide an explicit statement of the objective(s) or question(s) the review addresses. | 5 |
| **METHODS** | | |  |
| Eligibility criteria | 5 | Specify the inclusion and exclusion criteria for the review and how studies were grouped for the syntheses. | 5 |
| Information sources | 6 | Specify all databases, registers, websites, organisations, reference lists and other sources searched or consulted to identify studies. Specify the date when each source was last searched or consulted. | 5–6 |
| Search strategy | 7 | Present the full search strategies for all databases, registers and websites, including any filters and limits used. | 6, 27 |
| Selection process | 8 | Specify the methods used to decide whether a study met the inclusion criteria of the review, including how many reviewers screened each record and each report retrieved, whether they worked independently, and if applicable, details of automation tools used in the process. | 6 |
| Data collection process | 9 | Specify the methods used to collect data from reports, including how many reviewers collected data from each report, whether they worked independently, any processes for obtaining or confirming data from study investigators, and if applicable, details of automation tools used in the process. | 6 |
| Data items | 10a | List and define all outcomes for which data were sought. Specify whether all results that were compatible with each outcome domain in each study were sought (e.g. for all measures, time points, analyses), and if not, the methods used to decide which results to collect. | 6 |
|  | 10b | List and define all other variables for which data were sought (e.g. participant and intervention characteristics, funding sources). Describe any assumptions made about any missing or unclear information. | 6 |
| Study risk of bias assessment | 11 | Specify the methods used to assess risk of bias in the included studies, including details of the tool(s) used, how many reviewers assessed each study and whether they worked independently, and if applicable, details of automation tools used in the process. | N/A |
| Effect measures | 12 | Specify for each outcome the effect measure(s) (e.g. risk ratio, mean difference) used in the synthesis or presentation of results. | N/A |
| Synthesis methods | 13a | Describe the processes used to decide which studies were eligible for each synthesis (e.g. tabulating the study intervention characteristics and comparing against the planned groups for each synthesis (item #5)). | N/A |
|  | 13b | Describe any methods required to prepare the data for presentation or synthesis, such as handling of missing summary statistics, or data conversions. | N/A |
|  | 13c | Describe any methods used to tabulate or visually display results of individual studies and syntheses. | 6 |
|  | 13d | Describe any methods used to synthesize results and provide a rationale for the choice(s). If meta-analysis was performed, describe the model(s), method(s) to identify the presence and extent of statistical heterogeneity, and software package(s) used. | N/A |
|  | 13e | Describe any methods used to explore possible causes of heterogeneity among study results (e.g. subgroup analysis, meta-regression). | N/A |
|  | 13f | Describe any sensitivity analyses conducted to assess robustness of the synthesized results. | N/A |
| Reporting bias assessment | 14 | Describe any methods used to assess risk of bias due to missing results in a synthesis (arising from reporting biases). | N/A |
| Certainty assessment | 15 | Describe any methods used to assess certainty (or confidence) in the body of evidence for an outcome. | N/A |
| **RESULTS** | | |  |
| Study selection | 16a | Describe the results of the search and selection process, from the number of records identified in the search to the number of studies included in the review, ideally using a flow diagram. | 7 |
|  | 16b | Cite studies that might appear to meet the inclusion criteria, but which were excluded, and explain why they were excluded. | N/A |
| Study characteristics | 17 | Cite each included study and present its characteristics. | 9–11 |
| Risk of bias in studies | 18 | Present assessments of risk of bias for each included study. | N/A |
| Results of individual studies | 19 | For all outcomes, present, for each study: (a) summary statistics for each group (where appropriate) and (b) an effect estimate and its precision (e.g. confidence/credible interval), ideally using structured tables or plots. | N/A |
| Results of syntheses | 20a | For each synthesis, briefly summarise the characteristics and risk of bias among contributing studies. | 9–11 |
|  | 20b | Present results of all statistical syntheses conducted. If meta-analysis was done, present for each the summary estimate and its precision (e.g. confidence/credible interval) and measures of statistical heterogeneity. If comparing groups, describe the direction of the effect. | N/A |
|  | 20c | Present results of all investigations of possible causes of heterogeneity among study results. | N/A |
|  | 20d | Present results of all sensitivity analyses conducted to assess the robustness of the synthesized results. | N/A |
| Reporting biases | 21 | Present assessments of risk of bias due to missing results (arising from reporting biases) for each synthesis assessed. | N/A |
| Certainty of evidence | 22 | Present assessments of certainty (or confidence) in the body of evidence for each outcome assessed. | N/A |
| **DISCUSSION** | | |  |
| Discussion | 23a | Provide a general interpretation of the results in the context of other evidence. | 7–9 |
|  | 23b | Discuss any limitations of the evidence included in the review. | 19 |
|  | 23c | Discuss any limitations of the review processes used. | N/A |
|  | 23d | Discuss implications of the results for practice, policy, and future research. | 15–19 |
| **OTHER INFORMATION** | | |  |
| Registration and protocol | 24a | Provide registration information for the review, including register name and registration number, or state that the review was not registered. | N/A |
|  | 24b | Indicate where the review protocol can be accessed, or state that a protocol was not prepared. | N/A |
|  | 24c | Describe and explain any amendments to information provided at registration or in the protocol. | N/A |
| Support | 25 | Describe sources of financial or non-financial support for the review, and the role of the funders or sponsors in the review. | 22 |
| Competing interests | 26 | Declare any competing interests of review authors. | N/A |
| Availability of data, code and other materials | 27 | Report which of the following are publicly available and where they can be found: template data collection forms; data extracted from included studies; data used for all analyses; analytic code; any other materials used in the review. | N/A |

*From:* Page MJ, McKenzie JE, Bossuyt PM, Boutron I, Hoffmann TC, Mulrow CD, et al. The PRISMA 2020 statement: an updated guideline for reporting systematic reviews. BMJ 2021;372:n71. doi: 10.1136/bmj.n71. This work is licensed under CC BY 4.0. To view a copy of this license, visit https://creativecommons.org/licenses/by/4.0/

**Supplement S3: List of resources developed by the WHO that can support hospital AMS**

| - The WHO AWARE Antibiotic Book and Associated APP https://www.who.int/publications/i/item/WHO-MHP-HPS-EML-2023.04 - Essential Medicines list: https://www.who.int/groups/expert-committee-on-selection-and-use-of-essential-medicines/essential-medicines-lists - Antimicrobial stewardship programmes in health-care facilities in low-and middle-income countries: a WHO practical toolkit: https://iris.who.int/bitstream/handle/10665/329404/9789241515481-eng.pdf?sequence=1 - Health workers’ education and training on antimicrobial resistance: curricula guide. WHO & Public Health England 2019: https://apps.who.int/iris/bitstream/handle/10665/329380/9789241516358-eng.pdf - WHO competency framework for health workers’ education and training on antimicrobial resistance: https://iris.who.int/bitstream/handle/10665/272766/WHO-HIS-HWF-AMR-2018.1-eng.pdf?sequence=1 - WHO Policy Guidance on Integrated Antimicrobial Stewardship Activities: https://www.who.int/publications/i/item/9789240025530 - People-centred approach to addressing antimicrobial resistance in human health: https://iris.who.int/bitstream/handle/10665/373458/9789240082496-eng.pdf?sequence=1 - Open WHO AMS course Antimicrobial Stewardship: A competency-based approach English, Russian, French, Italian, Spanish, Mandarin https://openwho.org/courses/AMR-competency - Antimicrobial stewardship programmes in health-care facilities in low- and middle-income countries: a WHO practical toolkit: https://openwho.org/courses/practical-toolkit-for-AMS - Meaningful engagement of patients, survivors and carers in addressing antimicrobial resistance: https://www.who.int/publications/m/item/meaningful-engagement-of-patients--survivors-and-carers-in-addressing-antimicrobial-resistance |
| --- |

**Supplement S4: Interview—guiding questions**

**Country office - Interview questions:**

1. What have been your country’s major achievements in the development and roll out of programs on antibiotic stewardship and appropriate use?

2. What are some of the efforts conducted by the country office to support this work nationally?

3. Is there a national AMR focal point? Is there an established AMS committee? Do they meet regularly? When was their last meeting?

4. Can you name specific organizations or individuals who have had significant positive impact on the implementation of hospital AMS programs?

5. Are hospital AMS programs a requirement by local accreditation bodies?

6. Is there local dedicated training to AMS (beyond Infectious disease training)?

7. Is AWaRe integrated into any of the ongoing updates to medication use systems?

8. What facilitators and barriers have you encountered or do you foresee?

9. How can the WHO EMRO catalyze and facilitate this work?

**Organisations - Interview questions:**

1. What have been your major achievements in supporting the development and roll out of programs on antibiotic stewardship and appropriate use?

2. What is the scope and scale of your work? In particular, have you worked in countries of the Eastern Mediterranean region?

3. Are you aware of any specific initiatives (or individuals) that heavily support hospital antimicrobial stewardship efforts in EMR?

4. Are you interested in working in EMRO or doing more? If so, what facilitators and barriers have you encountered or do you foresee?

**Expert - Interview questions:**

1. What do you think have been the successes in addressing AMR and stewardship in your region?

2. What facilitators and barriers have you encountered or do you foresee?

3. What have been the most important learning from your experience in antimicrobial stewardship capacity building and program implementation?

4. Can you name specific organizations or individuals who have had significant positive impact on the implementation of hospital AMS programs?

5. What needs to happen to accelerate the roll out of effective stewardship?

6. How can WHO EMRO catalyze and facilitate this?

**Supplement 5: Regional and international organizations engaged in AMS and their key areas of expertise and engagement**

| **Organization** | **Policy** | **Advocacy** | **Implementation support** | **Education - online** | **Education – Face to face or hybrid** | **Mentorship** | **Technical support** | **Tools** | **Networking** | **Accreditation** | **Comments and Other** |
| --- | --- | --- | --- | --- | --- | --- | --- | --- | --- | --- | --- |
| Africa CDC | ✓ | ✓ | ✓ | ✓ | ✓ |  | ✓ | ✓ | ✓ |  |  |
| AMR Insights |  |  |  | ✓ | ✓ | ✓ |  |  | ✓ |  |  |
| British Society of Antimicrobial Chemotherapy (BSAC) | ✓ | ✓ |  | ✓ | ✓ |  |  |  |  |  |  |
| Global Antimicrobial Stewardship Accreditation Scheme (GAMSAS) |  |  |  |  |  |  |  |  |  | ✓ |  |
| Center for Infectious Disease Research and Policy (CIDRAP) |  | ✓ |  | ✓ |  |  |  |  |  |  |  |
| Commonwealth Pharmacists Association | ✓ | ✓ | ✓ | ✓ | ✓ | ✓ | ✓ | ✓ | ✓ |  |  |
| ESCMID Study Group for Antimicrobial Stewardship (ESGAP) |  |  |  | ✓ | ✓ | ✓ |  |  |  |  |  |
| Global Strategy Lab (GSL) - AMR policy Accelerator | ✓ |  |  | ✓ | ✓ |  |  |  |  |  | Evidence synthesis |
| International Center for Antimicrobial Resistance (ICARS) | ✓ | ✓ | ✓ | ✓ |  |  | ✓ |  |  |  | One Health Focus |
| International Pharmaceutical Federation (FIP) | ✓ | ✓ |  | ✓ | ✓ |  | ✓ |  | ✓ |  |  |
| IVPN network |  |  |  | ✓ |  |  |  |  | ✓ |  |  |
| Médecins Sans Frontières (Doctors without Borders) AMR division |  | ✓ | ✓ | ✓ | ✓ | ✓ |  |  |  |  |  |
| Merieux foundation |  |  |  | ✓ | ✓ |  |  |  |  |  | AMR and One health |
| Ministry of National Guard Health Affairs - WHO Collaborating Centre (WHOCC) for Infection Prevention and Control and Anti-Microbial Resistance |  |  |  |  | ✓  IPC and surveillance |  |  |  |  |  |  |
| National Centre for Antimicrobial Stewardship (NCAS) |  |  |  | ✓ |  |  |  | ✓ |  |  |  |
| Project ECHO* |  |  |  | ✓ |  |  |  |  | ✓ |  |  |
| Radboud University Medical Center (Dutch Antimicrobial Stewardship) |  |  | ✓ |  | ✓ | ✓ |  |  | ✓ |  |  |
| ReAct Africa |  | ✓ |  | ✓ | ✓ | ✓ |  | ✓ | ✓ |  |  |
| Stanford University - WHO CC for Antimicrobial Resistance and Stewardship |  |  | ✓ | ✓ | ✓ |  |  |  |  |  |  |
| Total number of organizations engaged in each key area | 6 | 8 | 6 | 16 | 13 | 6 | 4 | 4 | 8 | 1 |  |
| *Project ECHO were not approached for an interview but viewed as an important organisation that could in the future support EMR AMS scale up | | | | | | | | | | | |

**Supplement 6: Regional and international organizations engaged in AMS and their key areas of expertise and engagement: non-exhaustive experience summary**

| Theme | Example of relevant efforts from interviewed organizations |
| --- | --- |
| AMS programme systems and structure | In terms of scaling up implementation and tailoring policies, several organizations can provide support. **GSL for example could support policy development and** targeted evidence synthesis based on specific regional or national challenges. **ICARS, ESGAP, Radboud, Stanford University, and** **NCAS** could also support tailored intervention implementation. Of note, the **Africa CDC’s** North African Regional Coordinating Centre will soon be established and can support efforts in EMRO North African countries through training, networking, funding, technical and implementation support of AMS efforts highlighted in the Pan African treatment guidelines. Strengthening advocacy in the region for AMR and AMS scale-up could benefit from the experience of organizations such as **ReAct, CIDRAP, and MSF.** |
| Training and education | Stanford University: Supports WHO in the continuous development and refinement of educational curricula and programs, on AMS approaches and provision of high-quality services in clinical bacterial infectious disease management across the continuum of care.  The King Abdulaziz Medical City Hospital, Riyadh and the American University of Beirut Medical Center are the only WHO CC working on AMR in the region currently. |
| Mentorship and centers of excellence | Several organizations have experience with training coupled with one-to-one mentorship (MSF, ESGAP, Radboud, CPA) or hospital twinning (CPA) in which an AMS centre of excellence and an interested centre work together on strengthening AMS capacity team to team. |
| AMS tools | Some organizations, such as the **National Centre for Antimicrobial Stewardship (NCAS)**, focus on the development of electronic support tools for AMS with not only experience in use in Australia, but a variety of LMIC settings across Asia. The **Commonwealth Pharmacist Association (CPA)** has also developed tool kits available on their website that can be adapted and adopted. |
| Networking | Examples of networking platforms in the African context include those led by organizations such as ReAct Africa, the African Society for Laboratory Medicine, Africa CDC, and the Commonwealth Pharmacists Association’s network. Project ECHO provides a platform for information and dissemination across a range of subjects and has collaborated with WHO around HIV COVID19 and hepatitis. Some countries have involved professional societies that can facilitate such efforts but are often not dedicated to or focused on AMR or AMS. Networking not only allows peer to peer learning, but also sponsors research efforts and the identification and empowerment of local expertise necessary for the scale up efforts. Examples include the Lebanese Society of Infectious Diseases and Clinical Microbiology(71), La Société Tunisienne de Pathologie Infectieuse (STPI)(72), The Eastern Mediterranean Public Health network(73), all of which could work on AMR and AMS locally as part of their mandate. One regional organization called **Intravenous Parenteral Nutrition Network** (**IVPN)**(74) with over 27,000 members supports a network of pharmacists and physicians and provides continuous medical education regularly on various topics. It has a strong grassroot community which drives the educational sessions and organizes peer learning through focused listserv email groups.  The **International Pharmaceutical Federation (FIP)**(75)**, and AMR insights**(76) also have avenues for networking from members of the EMR community. In addition, the WHO at a global level supports a Community of Practice, and a regional window has been developed, but utilisation remains low. **Project ECHO** (Extension of Community health outcome)(77) is a facilitated platform for clinical dialogue that provides a hub and spoke model for dissemination of information and virtual support and discussion for clinicians and has been providing some sessions on AMR surveillance in conjunction with the African Society of Laboratory Medicine. |
| Accreditation and regulation | A few organizations, such as Global Antimicrobial Stewardship Accreditation Scheme (GAMSAS), or the Infectious Disease Society of America (IDSA) offer accreditation to hospitals worldwide. |

See Supplement S5 for amplification of institutional acronyms

**Supplement S7: Chosen quotations from interviews with experts organized per identified theme**

| Section Title | Quotes | Interviewee/speaker |
| --- | --- | --- |
| Theme 1: ASP systems and structure | In some countries like Jordan, Lebanon, and Egypt, pharmacists and clinical pharmacists are readily available and are a great resource | WCO AMR focal point |
|  | We need dedicated staff. Every month, we had a clear plan of action, but you lose people on the way because of other commitments. We need dedicated AMS staff, even if not full time | AMR official, WHO |
|  | Understanding models for healthcare delivery before implementing AMS systems is important for success and sustainability | Expert clinical pharmacist and senior policy advisor, Kenya |
|  | It is important to leverage the valuable expertise of nurses, pharmacists, and microbiologists in supporting and leading AMS | AMR expert, WHO Collaborating Center (WHO-CC) |
|  | AMS is tough and many end up burning out as you start many projects and there are many barriers, so having a champion dedicated to AMS with leadership support is important | Infectious diseases physician, policy maker |
|  | In our AMS upscaling efforts, we need to involve management from the start, or else success is very low” | International AMS/AMR policy expert |
|  | In many situations, pharmacists have been leading ASPs very well and in coordination with physicians. Pharmacists need to be empowered to lead AMS efforts by providing necessary training and visible advocacy by clinical champions and hospital leadership | Head of international NGO on AMR |
|  | It is important to change behaviour across the board regardless of speciality | Physician expert, Head of international society working on AMR/AMS |
|  | Everyone should feel responsible for rational antimicrobial use | Senior pharmacist leader, Ministry of Health |
| Theme 2: Training and education | One of the barriers is the lack of understanding of what they are supposed to do, but once they know (after training), they usually take up the interventions. Implementation is easier for the hospitals that have had training and mentorship, even in the absence of technical capacity | Expert clinical pharmacist and senior policy advisor, Kenya |
| Theme 2.1: Undergraduate or pre-service education | There is a need to introduce AMS and IPC ideas much earlier as core to patient safety and better outcomes, else these will be viewed as additional responsibilities | Head of international agency working on AMS/AMR |
| Theme 2.2: Specialised training on AMS program components, implementation, and leadership | There is a need to formalise AMS training for pharmacists | Physician and regional expert |
|  | It is crucial to train healthcare workers on AMS in teams such as nurses, prescribers, and pharmacists all together | Physician and international expert |
|  | The training needs to be practical and include communication and other soft skills | Regional AMS/AMR expert |
|  | There are differences in the culture, expectations, and in how people like to learn | Programme lead, International educator agency |
| Theme 3: Mentorship and centres of excellence | It was important to have long term clinical mentorship for the trainees to ensure their knowledge can be transferred to actions on daily tasks | Program manager, international organisation |
|  | The development of centres of excellence will help drive significant change in the country and region. One way to do this is through twinning of hospitals | Head of international agency working on AMR/AMS |
| Theme 4: AMS tools | Many hospitals don’t have tools to support the development of well-structured AMS programmes and don’t know about some of the available tools | Expert clinical pharmacist and representative for regional networking and training organisation |
|  | The available resources can be so confusing. It could be very helpful to categorise available resources and make them available in one platform for the users to have easy access | AMR/AMS regional expert |
|  | Some countries don't have antimicrobial use guidelines, and in some instances, it may be more useful to have “appropriateness criteria” as it is easier to implement and popularize | Senior official, WHO-CC |
|  | Simple electronic decision support tools can help implement AMS. They do not need to be comprehensive and embedded into electronic medical records if those are not available, but they can still have an impact on monitoring consumption, antimicrobial choice and dose optimization | AMR/AMS regional expert |
| Theme 5: Networking | There is great benefit in informal conversations and networking to ensure sustainability of AMS | Senior official, WHO CC |
|  | One of the biggest challenges in the region is the lack of networking and this is why we need to promote virtual networking activities | Expert clinical pharmacist and regional networking organization representative, United Arab Emirates |
|  | There are no dedicated resources for developing a regional platform for networking and collaboration, which is highly needed | Physician and regional expert |
| Theme 6: Accreditation and regulation | Local accreditation requirements as well as international requirements allowed us to gather support from leadership and other stakeholders for AMS implementation | Expert pharmacist, Egypt |
|  | Specialist AMS Training alongside regulation and accreditation may be more impactful that either intervention alone | AMR/AMS regional expert |
| Theme 7: Research | We need to study our population and understand how they think and behave when it comes to antimicrobial use and resistance. International data are very useful but often, local data are more impactful and appropriate to support decision making | Senior AMR expert |
